# Deterministic processes shape bacterial community assembly in a karst river across dry and wet seasons

**DOI:** 10.3389/fmicb.2022.938490

**Published:** 2022-10-06

**Authors:** Yongjie Wu, Yang Zhang, Xueqin Yang, Kaiming Li, Bixian Mai, Zhili He, Renren Wu

**Affiliations:** ^1^State Environmental Protection Key Laboratory of Water Environmental Simulation and Pollution Control, South China Institute of Environmental Sciences, Ministry of Ecology and Environment of the People’s Republic of China, Guangzhou, China; ^2^Environmental Microbiomics Research Center, School of Environmental Science and Engineering, Sun Yat-sen University, Guangzhou, China; ^3^State Key Laboratory of Organic Geochemistry and Guangdong Key Laboratory of Environmental Resources Utilization and Protection, Guangzhou Institute of Geochemistry, Chinese Academy of Sciences, Guangzhou, China; ^4^Guangdong-Hong Kong-Macao Joint Laboratory for Environmental Pollution and Control, Guangzhou Institute of Geochemistry, Chinese Academy of Sciences, Guangzhou, China; ^5^Southern Marine Science and Engineering Guangdong Laboratory (Zhuhai), Zhuhai, China

**Keywords:** karst river, spatiotemporal patterns, 16S rRNA gene amplicon sequencing, community assembly, external immigration

## Abstract

Karst rivers are particularly vulnerable to bacterial pollution because immigrations are easily diffused from the surrounding environments due to their strong hydraulic connectivity. However, the assembly mechanism in shaping riverine bacterial biogeography is still poorly understood, especially for an ecosystem in the karst area. Here, 16S rRNA genes were used to explore the spatiotemporal and biogeographical patterns of bacterial communities from the Chishui River in the dry and wet seasons, and explore the impact of external immigration on the assembly of water bacterial communities. Our results showed clear spatiotemporal patterns of bacterial communities with a more pronounced seasonal rather than spatial fluctuation, which appeared to be dependent on seasonal-related environmental factors (e.g., temperature and turbidity). The bacterial communities exhibited a significant (*p* < 0.05) distance–decay pattern in both seasons, and they had a stronger distance–decay relationship in the dry season than in the wet season. However, most of the biomarkers of different external immigrations did not show significant (*p* > 0.05) distance–decay patterns along the Chishui river, implying that the biomarkers could be used as indicators of external immigration (e.g., OTU_125 and OTU_536). Also, the tributaries were the main external immigration (20.44–83.68%) for the Chishui River, while other terrestrial immigration (e.g., livestock, the soil of the cropland, brewing wastewater treatment plant, and sewages) showed relatively little influence, which could be due to the hydrodynamic conditions (e.g., fragile rock–soil system and hydrological structure) of the karst river. Additionally, the assembly of water bacterial communities in the Chishui river was governed by more determinism (50.7–85.7%) than stochasticity (14.3–49.3%) in both the dry and wet seasons. We demonstrated that the bacterial community’s substantial variations are largely shaped by deterministic processes, thereby providing a better understanding of spatiotemporal patterns and mechanisms of the bacterial community in karst river waters.

## Introduction

Bacteria are fundamental components of ecosystems and play a vital role in global biogeochemical cycling (Ruiz-González et al., 2015; Kuypers et al., 2018; Fan et al., 2021). River bacterial communities are characterized by high-spatiotemporal variability caused by both the hydrology and external immigration, as continuous inflow of water from a wide range of external immigration allows a diverse pool of source communities to be assembled (Ruiz-González et al., 2015). It is well-known that knowledge about the composition and dynamics of bacterial communities across space and time is a prerequisite for predicting or manipulating river microbiomes (Read et al., 2015; Savio et al., 2015; Zhang et al., 2019). However, despite increased interest in the bacterial community assembly processes (Wu et al., 2018; Jiang et al., 2021; Liu et al., 2021; Zhang L. et al., 2021), we still lack an adequate understanding of how community assembly processes are influenced by external immigration.

Bacterial community assembly and the mechanisms of how different bacteria assemble into a community in aquatic ecosystems are still poorly understood, especially in river systems (Zhou and Ning, 2017; Zhang W. et al., 2018; Zhang L. et al., 2021). Bacteria have a high passive dispersal capacity and exhibit varying degrees of distance–decay patterns in the river systems (Zhang et al., 2014; Yan et al., 2018), but there was insufficient knowledge of how they dispersal along the river systems. The unified neutral theory suggested that microbial diversity arises *via* stochastic processes (e.g., birth, death, dispersal, and speciation) (Rosindell et al., 2011). Conversely, the niche theory claim that microbial communities are shaped by the deterministic processes due to habitat preferences and fitness of microbiomes, which include the biotic and abiotic factors (Rosindell et al., 2011; Stegen et al., 2013). However, previous studies evidenced a mixture of processes for the bacterial community assembly in river systems (Graham et al., 2017; Gweon et al., 2021; Zhang P. et al., 2021). For example, someone suggested that the bacterial community was largely shaped by deterministic processes in the world’s largest water diversion canal (Zhang P. et al., 2021), but another study showed that stochastic processes exhibited a greater influence on the bacterial assembly in the River Thames (Gweon et al., 2021). Environmental selection (deterministic process) plays an important role in dynamic habitats with pronounced environmental gradients, whereas stochastic processes could govern community assembly in less variable environments (Verberk, 2012). Therefore, further studies were needed on the assembly mechanism of river bacterial communities.

Ubiquitous in natural ecosystems, external immigration (e.g., pollution source) is one of the mechanisms shaping the community assemblage (Ruiz-González et al., 2015). However, most of such studies were focused on the effects of river water hydrodynamics on microbial communities, while their relationships with external immigration remain less explored (Read et al., 2015; Mei and Liu, 2019). It is important to characterize the rules governing the bacterial assembly processes along a river to understand the bacterial dynamics in response to external immigration (Mei and Liu, 2019). However, external immigrations are frequently reported in engineered water systems, it remains challenging to quantitatively address to what extent inputs contribute to the assembly of the river communities in the natural environment (Price et al., 2018; Ju et al., 2019), especially for karst rivers with complex environmental conditions.

Karst ecosystems account for about 10% of the Earth’s land, and 25% of people around the world depend on karst water resources, but the microbial ecology of karst river ecosystems has received less attention (Diston et al., 2018; Liang et al., 2018). Due to the carbonate rocks dissolve in karst ecosystems, fractures and other open spaces become enlarged, forming conduits, caverns, and sinkholes, inducing rapid drainage, and the karst aquifers are extremely permeable (Zhang R. et al., 2018). And karst groundwater is subjected to rapid surface recharge at points, the water resources are particularly vulnerable to pollution events (Diston et al., 2018). Especially, karst rivers are particularly vulnerable to bacterial pollution, and the healthy functioning of karst rivers is threatened because immigrations are easily diffused and spread from the surrounding environments due to their strong hydraulic connectivity (Xiang et al., 2021). Microbial communities could succeed during the transportation of external immigration into the river, which may highly depend on the time for water percolation into the river (Shabarova et al., 2013). However, microbial investigation in karst areas has been focused on the microbial community diversity, composition, isolation, and characterization of specific functional bacteria and pathogenic bacteria (Diston et al., 2018; Ender et al., 2018; Gu et al., 2020), we know little about the impact of external immigration on the assembly of bacterial communities of karst rivers.

The Chishui River with its unique karst landscape, the color of the water changes with the seasons, the river water is reddish in the wet season, but it becomes clear in the dry season. Chishui River has a total length of 436.5 km and a drainage area of 20,440 km^2^, it is not only the main source of drinking water in the Yunnan-Guizhou Plateau but also the well-known wine river in China due to the unique geological and landform environment. According to the longitudinal variations in altitude, the Chishui River basin is divided into three river sections: upstream (altitude: 1,000–1,930 m), midstream (500–800 m), and downstream (<500 m). The Chishui River is the only first-level tributary in the upper reaches of the Yangtze River without a dam, which microbial community is subject to potentially fewer human effects. Furthermore, karst rivers are highly vulnerable to chemical and biological contaminations and add more challenges to model microbial community dynamics, thus calling for a comprehensive investigation of microbial community patterns in the Chishui River for establishing an integrative assessment of water quality with microbial community monitoring (Sagova-Mareckova et al., 2021). Despite their important roles in controlling geochemical processes and indicating water quality (Anderson, 2018; Kuypers et al., 2018; Fan et al., 2021), little is known about the dynamic of bacterial community throughout a natural river of karst areas (Xiang et al., 2020).

In this study, we aimed to comprehensively understand the diversity, composition dynamics, and assembly of water bacterial communities along the Chishui River during dry and wet seasons, and address three scientific questions: (i) How does the Chishui River bacterial community vary at a spatial-temporal scale? (ii) How do the external immigrations impact the water bacterial community from the Chishui River? (iii) How important are the ecological processes in shaping the bacterial community assembly of the Chishui River? Our study contributes to a better understanding of spatiotemporal patterns of bacterial communities and their assembly mechanisms and has important implications for shed light on the underlying ecological processes and assembly mechanisms in a karst river.

## Materials and methods

### Site selection and sampling

In this study, 11 sample sites of water were taken along the Chishui River in the dry and wet seasons in March and September 2021, respectively. And four sites in the upstream and midstream, respectively, and three in the downstream (Supplementary Figure 1). Three water samples were collected from the top 5–10 cm layer using a stainless-steel core sampler at each site, and all samples were kept at 4°C and sent back to the laboratory within 3 h after collection. In the laboratory, each sample was subsequently divided into two subsamples: one was used for physicochemical analyses, and the other for bacterial community analyses. For the bacterial community analysis, 2 L of water sample was filtered through 0.22 μm polycarbonate filters (47 mm diameter, Whatman, Maidstone, UK), and the filters were stored at −80°C for further DNA extraction. Additionally, to explore the relative contributions of external immigrations to the bacterial community in the Chishui River, 166 samples of different external immigrations were collected during the dry season in March 2021. We sampled 33 livestock [the fresh fecal of pig (12), chicken (12), and cow (9)], 33 sediments of the Chishui River, 33 water of the tributaries, 27 soil of the cropland, 12 brewing wastewater treatment plant, and 28 sewages (wastewater treatment plants) along the whole basin for source-tracking analysis and stored with the filters under the same conditions (Supplementary Figure 1).

### Environmental variables analysis

Dissolved oxygen (DO), pH, conductivity, and temperature (T) were measured *in situ* with a multi-parameter water quality probe YSIEXO2 (Yellow Springs Instruments, USA), and the turbidity was measured at 1,900°C (HACH, China). Total nitrogen (TN), total phosphate (TP), and chemical oxygen demand (COD) of water samples were analyzed with previously described methods (Zhang Y. et al., 2021). Furthermore, the concentration of V, Cr, Mn, Co, Ni, Cu, Zn, Cd, Sb, Ba, Al, and Pb were simultaneously determined with an inductively coupled plasma mass spectrometry (ICP-MS, Agilent 7500 series, USA). In total, 19 environmental variables were measured in this study.

### Sequencing of 16S rRNA gene amplicons and data analysis

Extraction and purification of microbial community DNA from filtered water, sediment/soil, and fecal were carried out using the Power Water DNA Isolation Kit, Power Soil DNA Isolation Kit, and Power Fecal DNA Isolation Kit (MOBIO laboratories, Carlsbad, CA, USA), respectively. The quantity and quality of DNA were determined using a NanoDrop Spectrophotometer (NanoDrop Technologies Inc., Wilmington, DE, USA), and the highest quality DNA was used for library construction. A dual-index sequencing strategy was used to amplify the V3 and V4 regions of the bacterial 16S rRNA gene with universal primers 338F (50-ACTCCTACGGGAGGCAGCA-30) and 806R (50-GGACTACHVGGGTWTCTAAT-3). The amplification conditions were as follows: initial denaturation at 95°C for 5 min, followed by 30 cycles of 95°C for 30 s, 52°C for 30 s, and 72°C for 30 s, and ending with a final extension at 72°C for 10 min. Library quality was assessed with a Fragment Analyzer, and libraries were subjected to 250 bp paired-end sequencing on a HiSeq platforming Novogene Technologies Corporation (Tianjin, China).

Quality filtering and pre-processing of raw sequences were conducted on the Linux and Galaxy pipeline^1^ (Feng et al., 2017), which integrated all the necessary bioinformatic tools, and parameters of each process were previously described (Kong, 2011; Magoc and Salzberg, 2011; Edgar, 2013). In brief, the overlapped paired-end sequences were first assembled using QIIME (Quantitative Insights into Microbial Ecology), and poorly overlapped and low-quality sequences such as those with length <140 and moving-window (5 bp) quality score <20 were removed before further analysis. To effectively compare with previous research in the references, we applied the same method to generate operational taxonomy units (OTUs), and the final sequences were assigned to OTUs by UPARSE2 at a 97% similarity threshold. The generated OTU table including all the samples was rarefied to 37,296 reads per sample for subsequent analysis. Taxonomy classification in this paper was performed according to the SILVA (v138.1) database (Quast et al., 2013).

### Source-tracking and ecological process analysis

Source-tracking analysis was used to estimate the proportion of microbial taxa in the Chishui River that came from external immigrations, according to a Bayesian approach, which may imply the contribution of dispersal sources to community compositions (Knights et al., 2011; Comte et al., 2017). Samples from the Chishui River water in different sections were designated as sinks, and the external immigration samples from other pollution sources and tributaries were tagged as sources both in the dry season and wet season. To identify putative bacterial bioindicators in different external immigrations, linear discriminant analysis (LDA) and effect size (LEfSe) analyses were performed (Segata et al., 2011), and a heatmap was generated based on the relative abundance of the bioindicators using the AUTOMAP package.

To quantify the influence of ecological processes (e.g., drift, selection, and dispersal) on the water bacterial community from the Chishui River, the null model analysis was performed using a framework to classify community pairs into underlying drivers of deterministic processes (e.g., heterogeneous selection and homogeneous selection) and stochastic processes (e.g., homogeneous dispersal, dispersal limitation, and undominated) as previously described (Wu et al., 2020). First, the variation or turnover of both phylogenetic diversity and taxonomic diversity was first measured with the null model-based phylogenetic, tested the phylogenetic signal, whether it is significant, and then measured taxonomic β-diversity metrics: β-nearest taxon indices (β-NTI) and Raup-Crick (RC_*Bray*_). Second, β-NTI in combination with RC_*Bray*_ was used to quantify the ecological processes that influence the Chishui River water bacterial community composition on a spatiotemporal scale. Finally, if |β-NTI| > 2, the community turnover is governed by the heterogeneous or homogeneous selection, which indicated that the community was governed by a deterministic process. Pairwise comparisons with |β-NTI| < 2 were further subjected to RC_*Bray*_: the fraction of pairwise comparisons with |β-NTI| < 2 and RC_*Bray*_ < − 0.95 indicated the homogenizing dispersal impact; and the fraction of pairwise comparisons with |β-NTI| < 2 and RC_*Bray*_ > 0.95 indicated the dispersal limitation impact, both homogenizing dispersal and dispersal limitation mean to be governed by the stochastic process; the fraction of pairwise comparisons with |β-NTI| < 2 and |RC_*Bray*_| < 0.95 indicated the impact of the “non-dominant” fraction that any process mentioned above (Stegen et al., 2013; Zhou and Ning, 2017).

### Statistical analysis

To assess the variation in diversity measures along the Chishui River, α-diversity metrics (Chao1 and Shannon diversity) were computed, and ANOVA was performed using GraphPad Prism 7.0, and all statistical tests were considered significant at a *p*-value <0.05. Spearman’s rank correlation analysis and mantel test were used to show the effects of physiochemical properties on the bacterial diversity of the Chishui river water. To visualize the whole patterns of bacterial communities, we performed principal co-ordinates analysis (PCoA), and the community dissimilarities were tested by the analysis of similarity (ANOSIM) and permutational multivariate analysis of variance (PERMANOVA) using the VEGAN package in R. Distance–decay relationship, which provides a directional model for variations in β-diversity across spatial scales, was used to explore the bacterial community biogeographic patterns. Pairwise geographic distances between samples were calculated from the latitude and longitude coordinates using the “geosphere” library, and were plotted against the pairwise Bray–Curtis dissimilarities using the “ggplot2” package in R. A Spearman’s rank correlation between Bray–Curtis dissimilarities and geographic distances was calculated. Redundancy analysis (RDA) was performed to explore the relationships between bacterial communities and physicochemical variables. Before the RDA, we used a forward selection procedure to select local physicochemical variables using the “ordiR2step” function from vegan, and all non-significant (*p* > 0.05) variables were eliminated in further analyses. Additionally, variation partitioning analysis (VPA) was applied to determine the relative contribution of spatial, environmental factors, and heavy metals to the microbial communities in the R package (Liu L. et al., 2018). And the raw sequence data for this study have been deposited in the European Nucleotide Archive (ENA) at EMBL-EBI under accession number PRJEB49238.

## Results

### Geochemical characteristics

The environmental factors and heavy metals from the dry and wet seasons were summarized in Supplementary Figures 2, 3. Except for DO, all environmental factors showed significant (*p* < 0.05) differences both in the sampling seasons and sections, and on the contrary, there was no significant (*p* > 0.05) difference in concentrations of 11 heavy metals at a spatiotemporal. In general, the mean value of pH, temperature, turbidity, and TN were significantly (*p* < 0.05) higher in the wet season than those in the dry season. The turbidity showed the highest in upstream both in the dry season and wet season, and conversely, the temperature showed the lowest in upstream and increased along the river. Additionally, pH and conductivity showed higher at midstream in the dry season, and TP and turbidity showed the lowest in midstream in the wet season. However, little difference in heavy metals was observed in space (sections) or time (dry/wet season). Only Zn showed higher in the upstream than in the midstream, but Cd was on the opposite trend, being significantly (*p* < 0.05) higher in the downstream than in the upstream.

### Microbial diversity varied between dry and wet seasons

**FIGURE 1:**
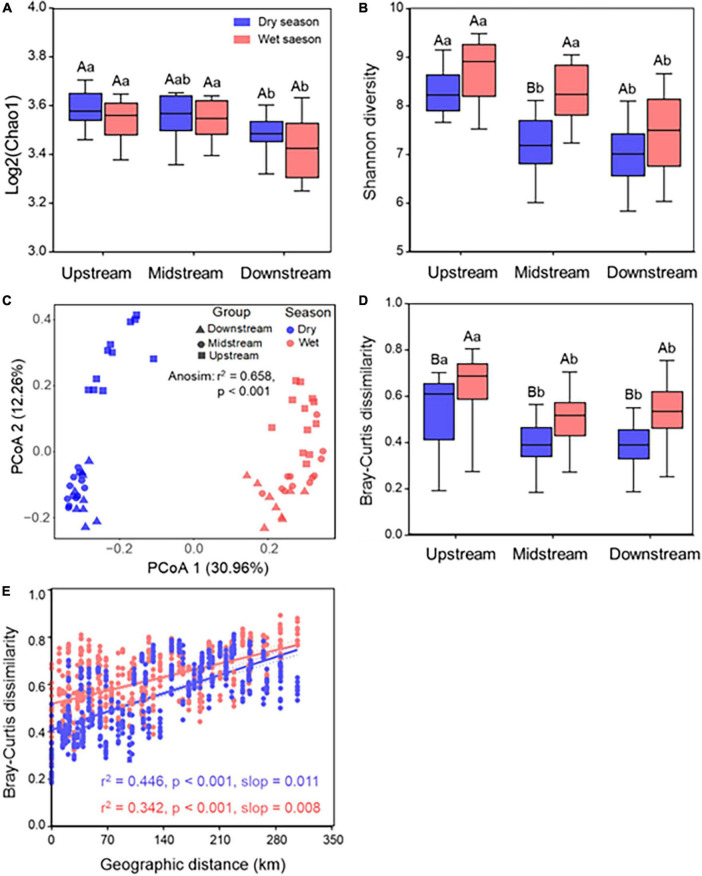
Bacterial biodiversity and distance–decay pattern of the Chishui River water. The Chao1 diversity **(A)** and Shannon diversity **(B)** of the Chishui River water between the dry and wet season along upstream, midstream, and downstream. ANOVA multiple comparisons were used to test the significance along different river sections. **(C)** Principal coordinated analysis (PCoA) based on Bray–Curtis distance and **(D)** respective Bray–Curtis dissimilarities; **(E)** pairwise relationships between Bray–Curtis dissimilarities and geographic distances for both in the dry and wet season samples, solid lines show linear regressions and dotted lines show 95% confidence interval of regression lines. Spearman’s rank correlations were calculated. The *p*-value was calculated by comparing the observed *F*-value with those from 1,000 randomized datasets. Different capital letters mean a statistical significance (*p* < 0.05) between dry and wet seasons within the same river section, and different small letters mean a statistical significance (*p* < 0.05) along different river sections within the same season.

A total of 10,866,884 sequences were obtained after filtering raw data of raw reads, which were clustered into 20,339 operational taxonomic units (OTUs). There was no significant (*p* > 0.05) difference in the Chao1 diversity along the Chishui River between dry and wet seasons; however, it showed a significant (*p* < 0.05) decrease in the downstream than in the upstream and midstream. The Shannon diversity was significantly (*p* < 0.05) higher in the wet season than in the dry season (Figures 1A,B). Additionally, the Shannon diversity was significantly (*p* < 0.05) higher in the wet season than in the dry season in the midstream, and significantly (*p* < 0.05) higher in the midstream than in the downstream in both dry and wet seasons. In general, the microbial community in upstream showed higher alpha diversity compared with those in the downstream in both dry and wet seasons. Furthermore, bacterial community β-diversity was compared among samples using a principal coordinate analysis (PCoA), and the first two principal components explained 43.22% of community variance based on the Bray–Curtis dissimilarity with a clear separation of communities both by river sections or by seasons (ANOSIM: *r*^2^ = 0.658, *p* < 0.001) (Figure 1C), and the PERMANOVA test further confirmed the significant difference (*p* < 0.001; Supplementary Table 1) between the dry season and wet season among different river sections. It implied that seasonal variation was more pronounced than spatial variation.

The bacterial composition varied both on spatial and temporal scales, and a stronger seasonal fluctuation was observed in river sections, which was consistent with PCoA analysis. *Proteobacteria* (21.54–60.72%), *Actinobacteria* (7.25–38.58%), *Firmicutes* (4.84–15.24%), *Bacteroidetes* (3.53–4.87%), and *Cyanobacteria* (0.87–2.93%) were the most abundant phyla in the Chishui River water (Supplementary Figure 4). At the genus level, the variation of bacterial composition was far more different than that at the phylum level both on spatial and temporal scales (Supplementary Figure 5). *Limnohabitans* (1.65–10.96%), *Acinetobacter* (0.84–8.39%), *Aurantimicrobium* (0.51–11.07%), *Exiguobacterium* (0.28–9.75%), *Uncultured-Acidimicrobiia* (0.53–7.63%), and *Pseudorhodobacter* (0.96–4.96%) were the most abundant genera (Supplementary Figure 5), mainly affiliated with *Proteobacteria*, *Actinobacteria*, and *Firmicutes* phyla. For example, the abundance of *Limnohabitans*, *Aurantimicrobium*, *Acinetobacter*, *Uncultured-Acidimicrobiia*, and *Pseudorhodobacter* in the midstream and downstream showed significant increase (*p* < 0.001) in the dry season than in the wet season; for the midstream, *Sphingorhabdus* and *Novosphingobium* were significantly (*p* < 0.001) higher in the wet season than in the dry season (Supplementary Figure 6).

To provide a directional model for variations in β-diversity variations across spatial scales, the distance–decay relationship was used to indicate a decreasing community similarity with increasing geographic distance. It showed that bacterial β-diversity was significantly (*p* < 0.001) higher the in the wet season than in the dry season all along the Chishui River, and it was significantly (*p* < 0.001) higher in the upstream than in the midstream or downstream both in the wet season than in the dry season (Figure 1D). Additionally, Spearman’s rank correlations between the Bray–Curtis similarity of river bacterial communities and spatial distances showed a significant (*p* < 0.001) distance–decay relationship both in dry and wet seasons (Figure 1E). Also, the correlations between spatial distances and bacterial communities exhibited a stronger (slop: 0.011 vs. 0.008) distance–decay pattern in the dry season than in the wet season.

### Contributions of the external immigration to water bacterial communities

**FIGURE 2:**
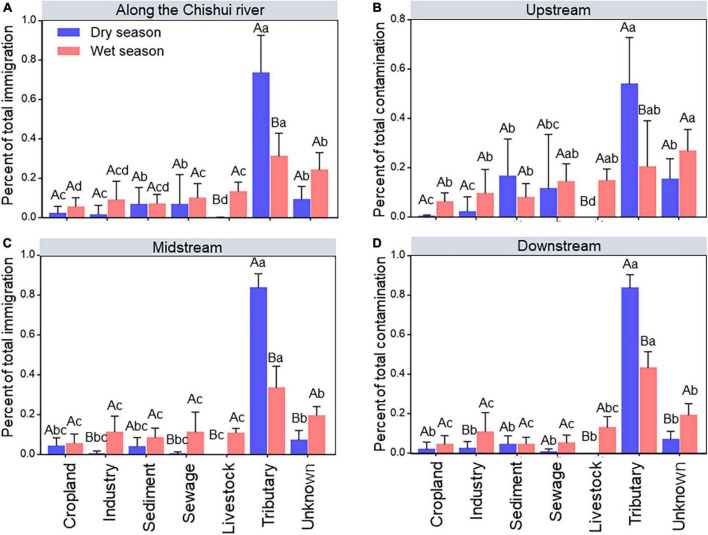
Source–tracking analysis showing the relative contributions of external immigrations to the overall microbial community at the whole Chishui River scale **(A)**, and the upstream **(B)**, midstream **(C)**, and downstream of the river **(D)** between the dry and wet season. “Unknown” includes the percentage of contributions not able to be attributed to a specific source. Different capital letters mean a statistical significance (*p* < 0.05) between dry and wet seasons within the same river section, and different small letters mean a statistical significance (*p* < 0.05) along different river sections within the same season.

To quantify the impact of the external immigrations contributed to the Chishui River community, source-tracking analysis was used to compare the external immigrations contributing to the river bacterial community between the dry season and wet season, respectively. It showed that the bacterial composition of the Chishui River was largely sourced from tributary (20.44–83.68%) and unknown (6.89–24.14%) immigration, and the value of tributary was significantly (*p* < 0.05) higher in the dry season than in wet the season, but the unknown proportion showed an opposite trend (Figure 2). It is interesting that livestock, even other immigrations had little or no impact on the Chishui River in the dry season. Also, the impact of external immigration on the bacterial communities in the midstream and downstream varied with these in the upstream, where the contribution of tributary was much lower (53.9%) than midstream (83.68%) and downstream (83.59%), and the contribution of sediment (16.62%) and sewage (11.5%) higher than in midstream and downstream in the dry season (Figure 2).

We further explored the specific bacterial taxa of different external immigrations by a LEfSe analysis aimed to explain any potential biomarkers. For each external immigration, the cropland, tributary, sewage, sediment, livestock, and industry had 1, 1, 7, 5, 2, and 1 potential biomarkers (LDA score ≥3) (Supplementary Figure 7), respectively. The biomarkers in the Chishui River varied both on spatial and temporal scales, and a stronger seasonal fluctuation was observed in river sections, which was also consistent with the whole bacterial community pattern. Although the variation in seasons of biomarkers is greater than the difference between river sections, most biomarkers were not significantly different along the Chishui River, and only a few have significant (*p* < 0.05) differences in different river sections, such as OTU_5 and OTU_22. And the most biomarker showed no significant (*p* > 0.05) difference among different river sections, such as OTU_125, OTU_120, OTU_536, OTU_13335, OTU_6979, OTU_34, and OTU_8751. It may possibly suggested that these biomarkers to be a potential indicator of external immigrations.

### Deterministic processes governed the water bacterial community assembly

**FIGURE 3:**
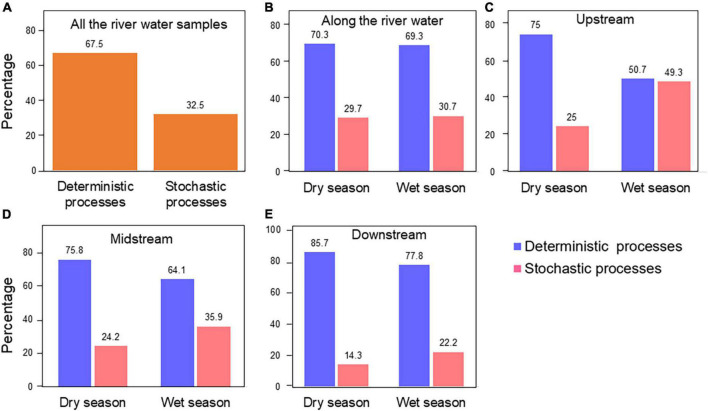
The summary contribution of ecological processes that govern the assembly of the microbial communities of all the Chishui River samples **(A)**, at the whole Chishui River scale **(B)**, upstream **(C)**, midstream **(D)**, and downstream **(E)**. The percentages (numbers on the individual bars) are given the relative contribution of the deterministic and stochastic processes to the community succession at different river sections.

To explore mechanisms underpinning the observed spatiotemporal ecological pattern of bacterial communities in the Chishui River, the relative roles of ecological processes for bacterial community assembly were quantified. Phylogenetic Mantel correlogram showing significant phylogenetic signal across short phylogenetic distances (Supplementary Figure 8). The assembly of bacterial communities from the Chishui River was largely driven by deterministic processes (67.5%), and stochastic processes (32.5%) (Figure 3A). Except that the wet season in the upstream, the deterministic (50.7%) and stochastic (49.3%) processes were similar between the dry and wet seasons. The bacterial community structure was largely shaped by the deterministic process (64.1–85.7%) in both dry and wet seasons for the midstream, downstream, and the whole river (Figures 3B–E). Additionally, the deterministic process exerted an increasing role along the river in both dry and wet seasons, and conversely, the stochastic process exerted a decreasing role. Additionally, homogeneous selection exerted a greater role in the dry season but heterogeneous selection exerted a greater role in the wet season, and homogeneous selection exerted the greatest role in the upstream (45.3%), then midstream (10.6%) and downstream (0.0%) in the dry season (Supplementary Figure 9). Overall, the results suggested that deterministic processes explained a higher proportion of the Chishui River bacterial community variation than stochastic processes, and the deterministic process was slightly higher in the dry season than in the wet season.

### Drivers of bacterial community structure between dry and wet seasons

**FIGURE 4:**
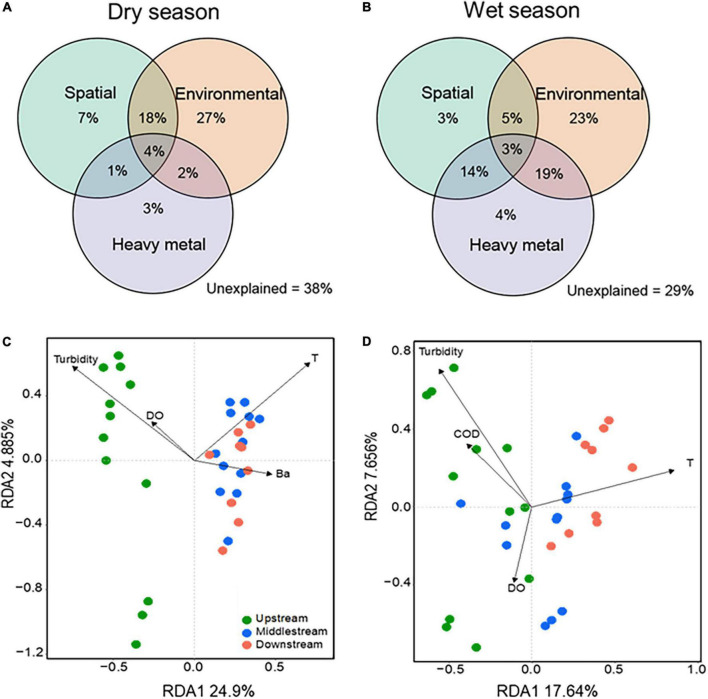
Drivers of microbial communities. The variation partitioning analysis (VPA) diagrams show the percentage contribution of spatial (river section, latitude, and longitude), environmental factors (pH, Conductivity, T, Turbidity, DO, TN, TP, and COD), and heavy metal (V, Cr, Mn, Fe, Co, Ni, Cu, Zn, Cd, Sb, and Ba) to bacterial community variations in the dry **(C)** and wet **(D)** seasons. The redundancy analysis (RDA) biplots show factors impacting bacterial communities in the dry **(A)** and wet **(B)** seasons. Dot color indicates samples from different river sections: green, upstream; blue, midstream; red, downstream. Only six factors identified to significantly influence bacterial communities are shown in the RDA plots. *T*, temperature; COD, chemical oxygen demand; DO, dissolved oxygen.

**FIGURE 5:**
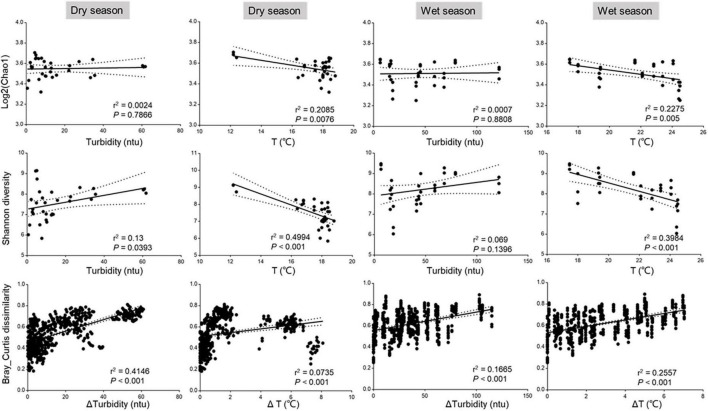
Linear regression analysis of correlations between the Chao1 diversity, Shannon diversity, and Bray–Curtis dissimilarity of bacterial communities and key environmental factors. Chao1 diversity values were log-transformed.

To better quantify the relative contributions of environmental factors to the variation of bacterial community structure, we conducted a variance partitioning analysis (VPA) on three subgroups of factors (i.e., spatial, environmental factors, and heavy metal) for dry and wet seasons, respectively. As shown in Figures 4A,B, the pure spatial contributed more in the dry season (7%) than in the wet season (3%), the pure environmental showed a consistent trend (27 vs. 23%), and the heavy metal showed a small contribution to the microbial variation in both dry (3%) and wet (4%) seasons. Also, the proportion of the variation in the dry season (38%) was higher than that in the wet season (29%) across all samples could not be explained by model parameters or by interactions between them, implying that more comprehensive and complex factors affect the structure and assembly of bacterial communities in the dry season than in the wet season. We further conducted the seasonal and spatial variation of drivers that appeared based on the RDA plots (Figures 4C,D). For instance, turbidity and temperature were the main drivers of the bacterial assembly in both dry season and wet seasons, which was supported by the significant correlations of the physiochemical properties with the microbial diversity by one-way ANOVA (Supplementary Table 2). Turbidity, temperature, DO, and Ba appeared to be an important factor for the bacterial community in the dry season; turbidity, temperature, COD, and DO largely impact the bacterial community structure in the wet season. To better understand the impact of drivers on the variation of microbial diversity, we also analyzed the linear regression of correlations between bacterial diversity and key environmental factors. Chao1 and Shannon diversity were significantly (*p* < 0.05) decreased with increasing temperature in both dry and wet seasons; Bray–Curtis dissimilarity significantly (*p* < 0.05) increased as turbidity increased but significantly (*p* < 0.05) decreased as temperature increased in the dry season, but an opposite trend was observed in the wet season (Figure 5). The results were consistent with the ecological process analysis that the deterministic process contributed more in the dry season than in the wet season, and indicated that turbidity, temperature, DO, and COD were the main drivers of bacterial community structure for the Chishui River water.

## Discussion

Understanding the ecosystem assembly mechanism is one of the most central goals of ecology. In this study, we examined the biogeography pattern of the bacterial communities from the Chishui River during the dry and wet seasons and evaluated the contribution of external immigration to the karst river. Furthermore, we provided a quantitative assessment of ecological processes governing bacterial community assembly at the spatiotemporal scale. We found that the diversity of bacterial communities decreased along the upstream to downstream, and the heterogeneous selection and homogeneous selection appeared to be the major forces for shaping the bacterial community. The tributaries were the main external immigration, and the proportion of other pollution diffused to the Chishui River was low, which may be related to the karst landform conditions. Our results indicated that the bacterial communities were assembled into a distinct community along the Chishui River, which was largely driven by seasonal-related environmental factors, such as turbidity and temperature.

The biogeography of bacterial communities across a long river still lacked attention in river ecosystems, especially for a karst river (Zhang W. et al., 2018; Zhang P. et al., 2021). A number of recent catchment-scale studies have highlighted that bacterial communities can exhibit a predictable gradient in the community composition from the headwaters to the downstream river, driven by the relative importance of terrestrial inputs and species sorting along the river continuum (Read et al., 2015; Savio et al., 2015; Henson et al., 2018). We observed that the upstream bacterial community had the highest diversity, which is consistent with previous studies (Savio et al., 2015; Wang et al., 2018). Several reasons may explain this phenomenon. First, it may be that the large contact zone of small headwaters with the surrounding environment facilitates the contribution of allochthonous bacteria to the river community (Besemer et al., 2013); also, these source environments of inoculation contain a much higher diversity than aquatic communities (Henson et al., 2018). Additionally, it may be the higher turbidity in the upstream, which has demonstrated that bacteria attached to suspended particles could constitute as much as 90% of total bacterioplankton production in riverine systems (Crump and Baross, 2000). It may possibly reflect the relative importance of immigrations or hydrological conditions in structuring these communities (Ruiz-González et al., 2015). Although external immigrations had played a vital role in structuring river bacterial, it is not clear which members are simply “passing through” without playing important roles in ecosystem functioning and which members are proliferating and adapted to the freshwater niche (Gweon et al., 2021); thus further studies are necessary to explore the influences of the external immigrations on the function of the bacterial communities along a river.

Planktonic communities are generally expected to be heterogeneous in both space and time, as continuous inflow of water from a wide range of external immigration allows a diverse pool of source communities to be assembled (Ruiz-González et al., 2015). Although external immigration has proven that it can influence the assembly of microbial communities in receiving ecosystems, it is frequently reported in engineered water systems, it remains challenging to quantitatively address what external immigrations contribute to the bacterial community assembly in natural environments (Mei and Liu, 2019). As expected, the tributaries were the overwhelming contribution of the dispersal source to the Chishui River, and the other external immigrations may have a limited dispersal to the river. This was inconsistent with the previous studies, which showed that livestock, domestic sewage discharge, and agriculture were the main contributors of river pollution (Liu G. et al., 2018; Lee et al., 2021). This discrepancy might be explained by the Chishui River is located in a karst area, and the external immigrations cannot reach the Chishui River due to that the immigrations may infiltrate into the groundwater quickly. Additionally, floods, rain storms, and water column mixing could largely increase the rates of immigrations dispersal into the river in the wet season, and the relative importance of different dispersal sources is expected to be higher than those in the dry season. And further studies were needed to explore the impact of external immigration on the karst river systems in combination with the special hydrological conditions and groundwater environment in karst areas.

It has been widely accepted that there is a distance–decay pattern in the water microbiome (Savio et al., 2015; Stanley et al., 2016); however, few studies have attempted to interpret the microbial distance–decay pattern from the perspective of quantifying the underlying process. Environmental selection and dispersal processes are the main drivers that influence the distance–decay pattern when communities are surveyed at large spatial scales (Wu et al., 2018). Although the bacterial community exhibited a similar biogeographic pattern in the dry season and wet seasons, distance–decay was more obvious in the dry season than in the wet season. The Chishui River bacteria exhibited a more distinct community compositions at a seasonal scale than at a spatial scale, which is consistent with the previous studies (Chen et al., 2019; Wang et al., 2021). Several reasons may explain this phenomenon. First, the river ecosystems are uniquely characterized by constantly mixing water, and the opportunities for the exchange of taxa from upstream to downstream are high (Gweon et al., 2021). Second, compared with the midstream and downstream sections, the upstream section is frequently disturbed. Additionally, it may be due to the environment related to the altitude among upstream, midstream, and downstream. These results indicated the importance of species sorting and dispersal limitation in shaping the Chishui River water bacterial community. However, most of the biomarkers did not follow a distance–decay pattern as clearly as the community along the river, it may due to that the biomarkers may vary in their dispersal efficiency. For example, the ability to survive during dispersal (Eichmiller et al., 2014; Mattioli et al., 2017). Additionally, dispersed taxa appear to have different abilities to colonize a new environment, for instance, some taxa may compete with indigenous for new carbon sources in the river (Adams et al., 2014). It was suggested that the valuable assessing the biomarkers for water monitoring and their potential in indicating ecosystem disturbance.

It is widely acknowledged that environmental selection and dispersal-related processes are two primary ecological processes determining the biogeographical distribution of bacterial communities (Chen et al., 2019; Liu et al., 2020; Gweon et al., 2021). However, given the complexity and variability of the natural environment, it remains challenging to quantitatively evaluate the contribution of this process to the microbial diversity in the receiving ecosystems (Mei and Liu, 2019). In our study, determinism rather than stochasticity had a greater impact on the Chishui River bacterial community assembly both in the dry season and wet seasons. However, this finding was inconsistent with previous studies, which showed that stochasticity influences were dominated by the river microbial community (Chen et al., 2019; Gweon et al., 2021). This discrepancy may be attributed to the fact that these studies were reported in plains river systems (Chen et al., 2019; Gweon et al., 2021), and the Chishui River is a karst river with a large altitude range (200–1,930 m). It should be noted that the consistent bacterial diversity patterns in the dry and wet seasons were not surprising, and likely reflect niche differences related to effects of seasonal-related environmental factors such as turbidity, temperature, DO, food resources, and availability of competitor or predator-free space. Subsequently, the variation of α-diversity we observed could easily reflect a species filtering effect as organisms disperse further from river influents. Different from the wet season, the bacterial community in the dry season had a slightly stronger response to spatial factors, which may be due to that the bacterial community occupies a greater variety of ecological niches in the dry season (Liao et al., 2016; Jiao et al., 2017). In summary, this study demonstrated that deterministic processes are largely in shaping substantial variation for the bacterial community along the Chishui River, thereby providing a better understanding of spatiotemporal patterns and mechanisms of the bacterial community in such karst river waters.

## Conclusion

This study provides a comprehensive understanding of the spatiotemporal patterns and assembly mechanisms underlying the bacterial community and reveals the importance of the deterministic processes on the bacterial community assembly in a karst river. It demonstrated that different seasons or river sections had distinct bacterial community compositions, and with a more pronounced seasonal rather than spatial fluctuation. The tributaries were an overwhelming contribution (20.44–83.68%) of dispersal source to the Chishui River, indicating that there was little direct influence of the other external immigrations on the Chishui River, which could be due to the hydrodynamic conditions (e.g., fragile rock–soil system and hydrological structure) of the karst river. However, most of the biomarkers of different immigration sources did not show significant (*p* > 0.05) distance–decay patterns along the Chishui River, implying that the biomarkers could be used as indicators of external immigrations. To fully understand the bacterial community assembly mechanisms in karst river ecosystems, it is suggested that future microbial community ecology researches should consider possible explanatory factors (e.g., species function and species interactions), and more other hydrology of karst landforms effects (e.g., the groundwater and fragile rock–soil systems).

## Data availability statement

The datasets presented in this study can be found in online repositories. The names of the repository/repositories and accession number(s) can be found in the article/Supplementary material.

## Author contributions

RW, KL, and BM conceptualized and supervised the study. RW and KL contributed to the funding acquisition and project administration. YW, YZ, and ZH investigated the study and performed the resources. YW, YZ, and XY contributed to the formal analysis. YW, XY, ZH, and BM visualized the manuscript. YW wrote the original draft of the manuscript. All authors contributed to the data analysis and interpretation and commented on the manuscript.

## Footnotes

1
http://mem.rcees.ac.cn:8080/
