# Supplementary material for: Deterministic processes shape bacterial community assembly in a karst river across dry and wet seasons
Source: Front Microbiol. 2022 Oct 6;13:938490. doi: 10.3389/fmicb.2022.938490 (PMC9584624; doi:10.3389/fmicb.2022.938490)
Supplement: Supplementary file 1 [file Data_Sheet_1.docx]

**Supplementary Figures**

**Supplementary Figure 1** Map showing the sampling sites along the Chishui River. Colors represent different sample types and as follows: Red: river water and sediment; Green: tributary; Orange: livestock; Violet: Sewage; Gray-black: industry; Grey blue: Cropland.


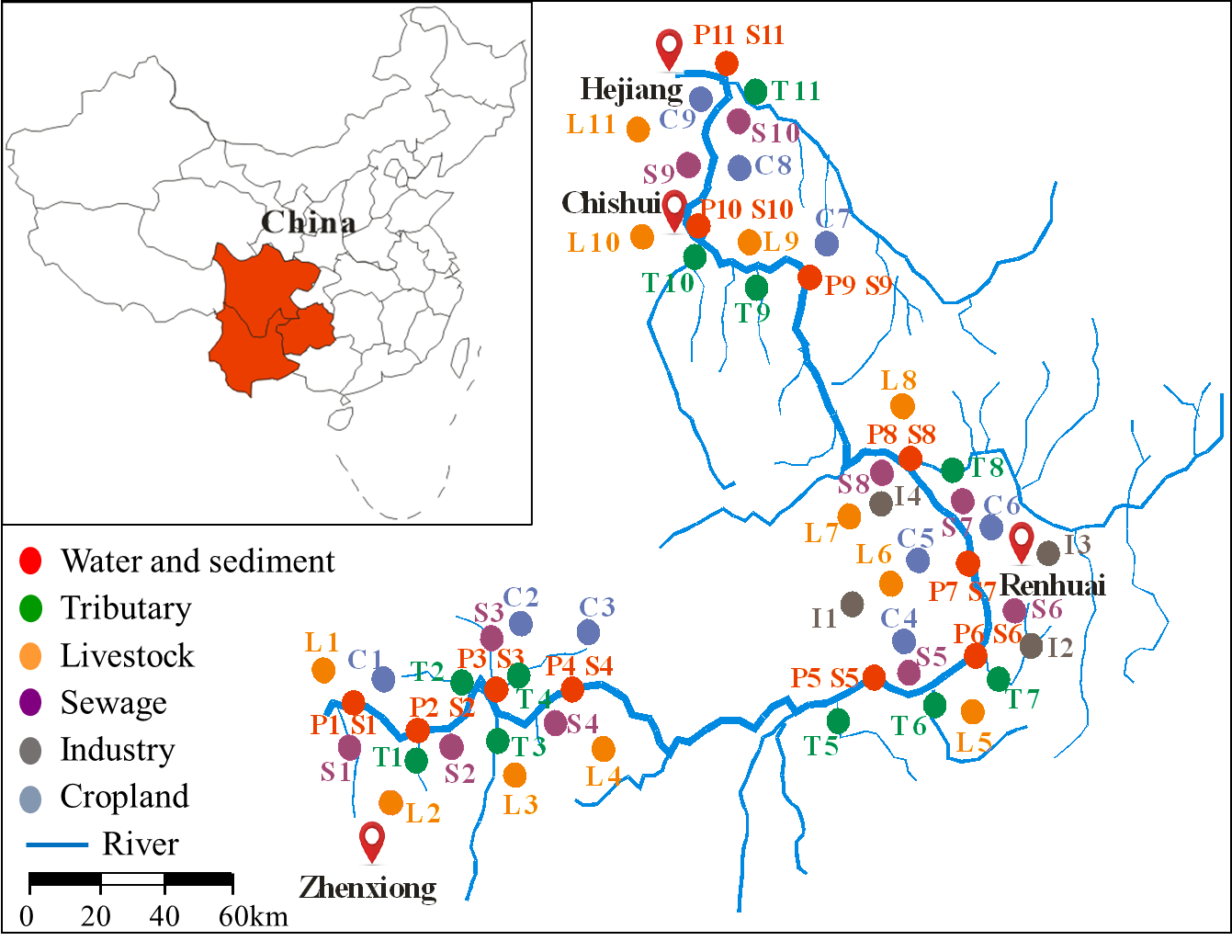


**Supplementary Figure 2** Environmental factors of Chishui river water between dry and wet seasons. A: pH; B: Conductivity; C: T (temperature); D: Turbidity; E: DO (dissolved oxygen); F: TN (total nitrogen); G: TP (total phosphorus); H: COD (chemical oxygen demand). Different capital letters mean a statistical significance (p < 0.05) between dry and wet seasons within the same site, and different small letters mean a statistical significance (p < 0.05) among different sites within the same season.


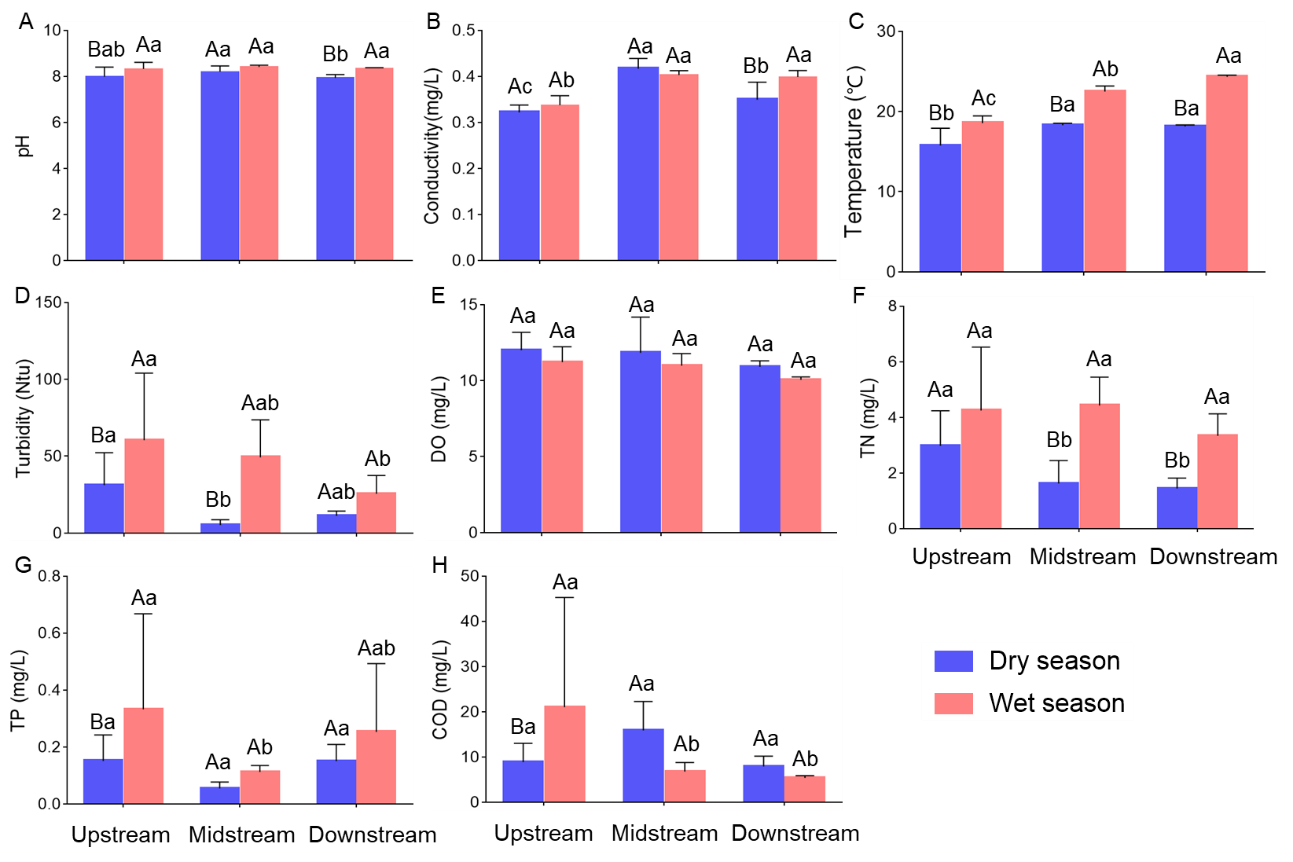


**Supplementary Figure 3** Heavy metals of Chishui River water between dry and wet seasons. A: V (vanadium); B: Cr (chromium); C: Mn (manganese); D: Fe (iron); E: Co (cobalt); F: Ni (nickel); G: Cu (copper); H: Zn (zinc); I: Cd (cadmium); J: Sb (stibium); K: Ba (barium). Different capital letters mean a statistical significance (p < 0.05) between dry and wet seasons within the same site, and different small letters mean a statistical significance (p < 0.05) among different sites within the same season.


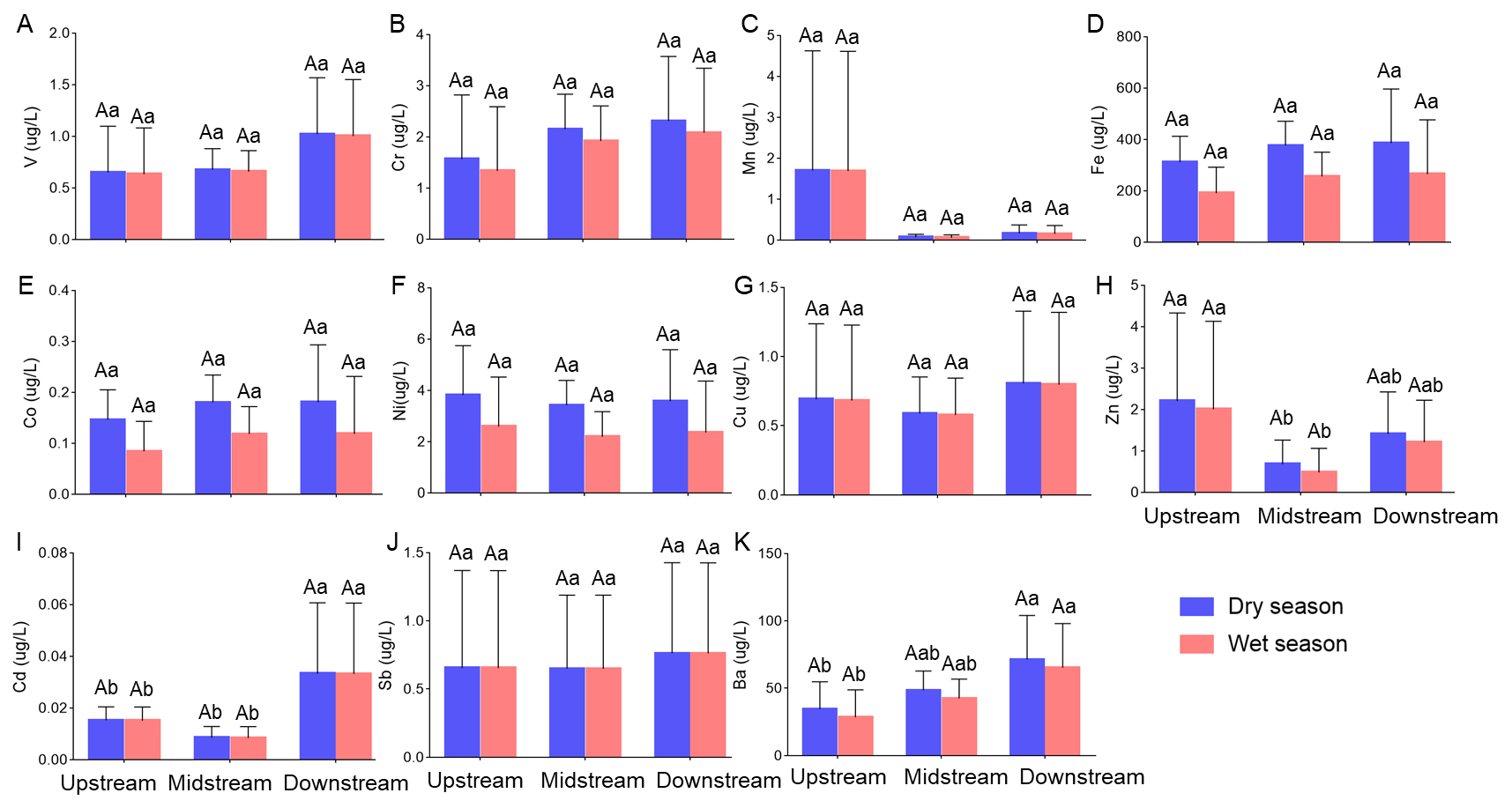


**Supplementary Figure 4** Taxonomic composition of microbial communities of Chishui River at the phylum level in dry season(A) and wet season (B) among different river sections. Different colors refer to different phyla.


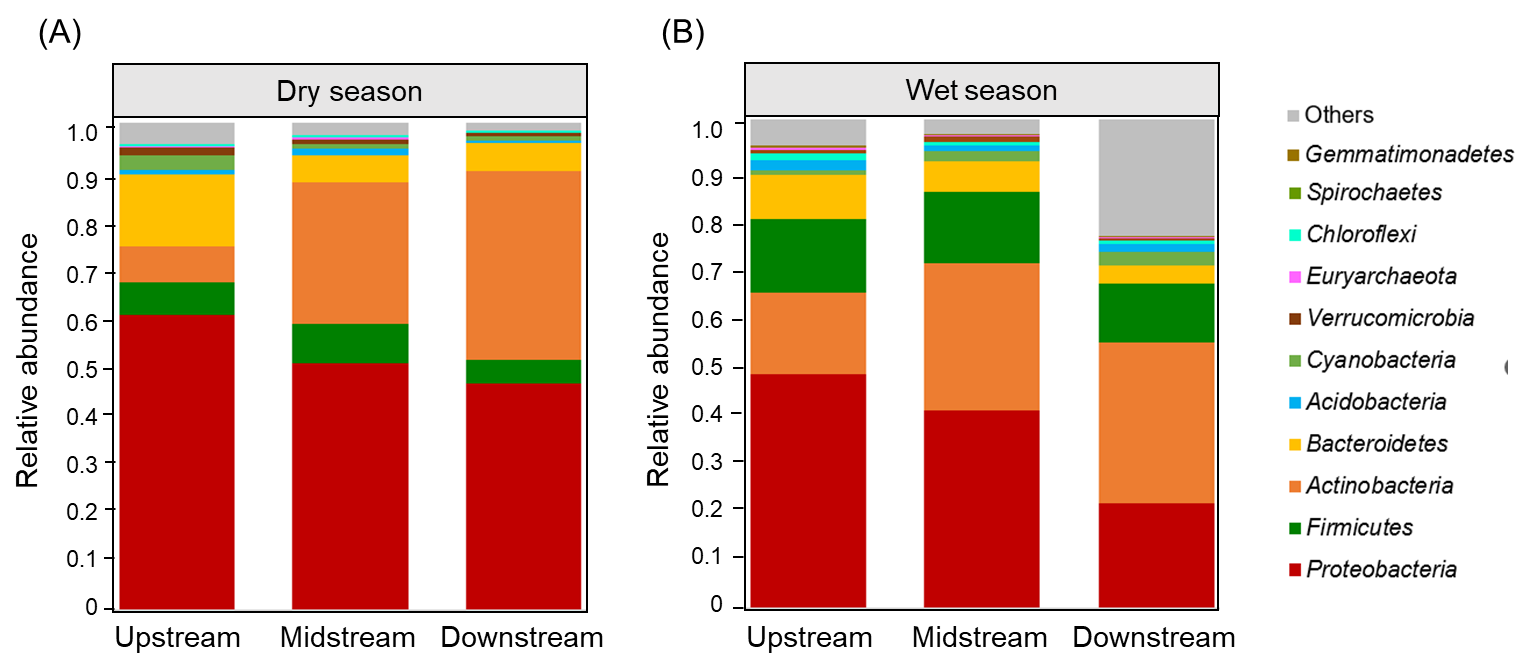


**Supplementary Figure 5** Taxonomic composition of microbial communities of Chishui River at the genus level in dry season (A) and wet season (B) among different river sections. Different colors refer to different genera.


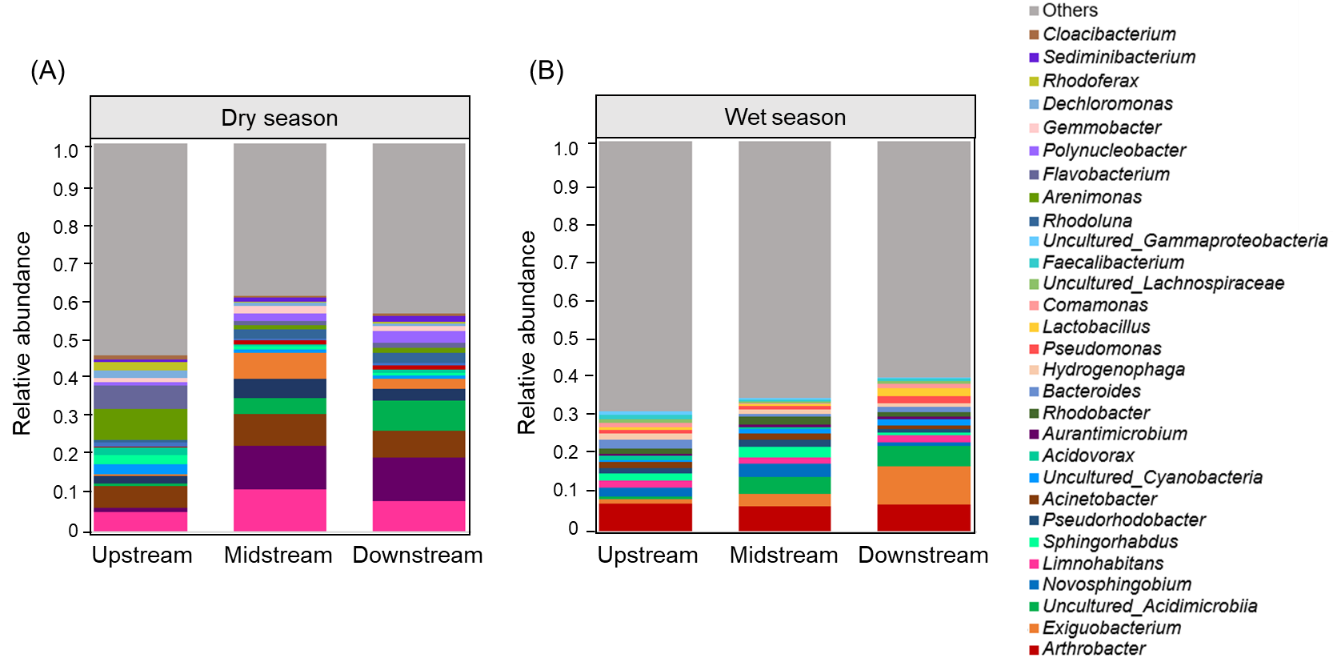


**Supplementary Figure 6** Relative abundances of dominant genera between dry and wet seasons in different river sections. Different capital letters mean a statistical significance (p < 0.05) between dry and wet seasons within the same site, and different small letters mean a statistical significance (p < 0.05) among different river sections within the same season.


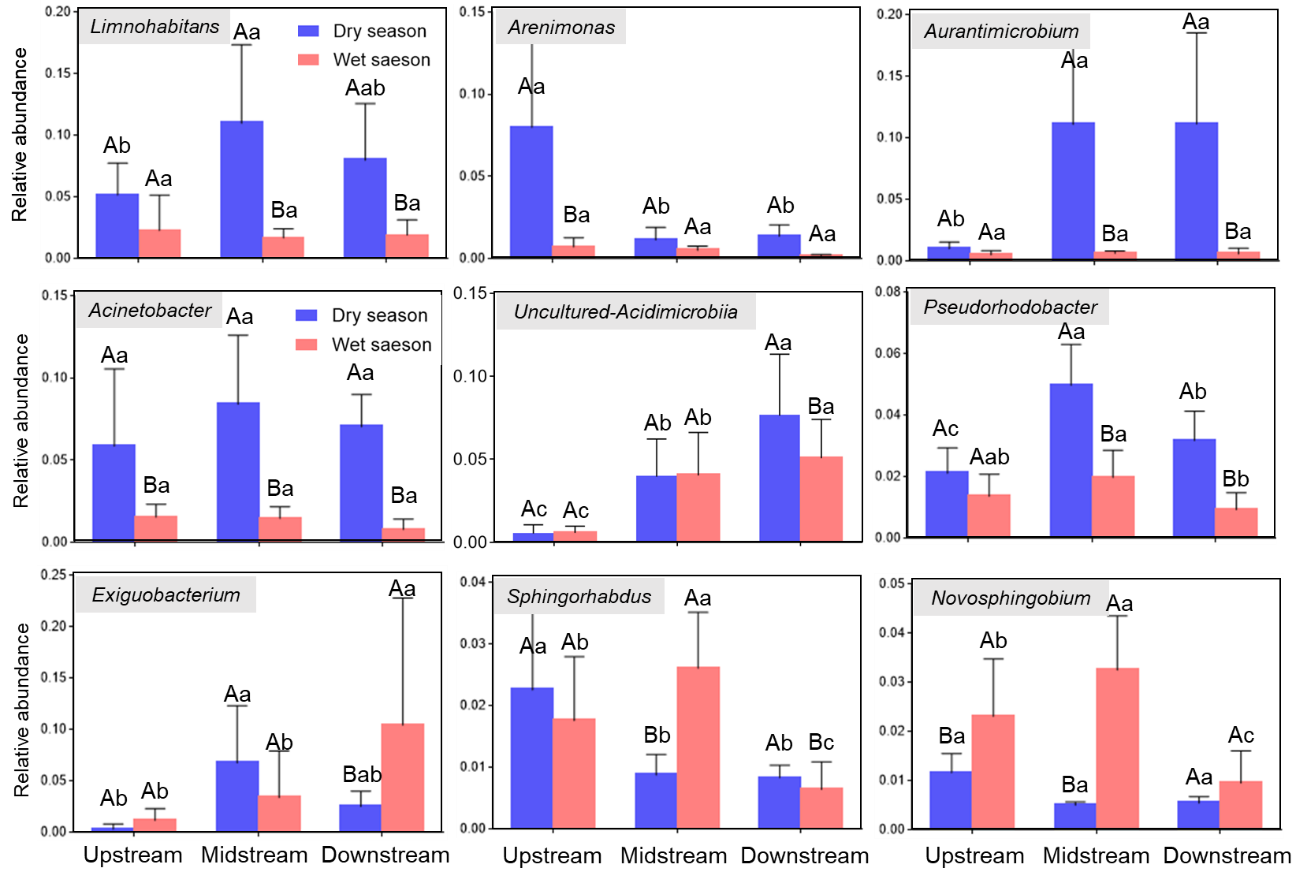


**Supplementary Figure 7** The biomarker of different external immigrations of Chishui River, as identified using linear discriminant analysis (LDA) effect size (LEfSe) (A), and the relative abundances of biomarkers along the river in the dry season and wet season (B).


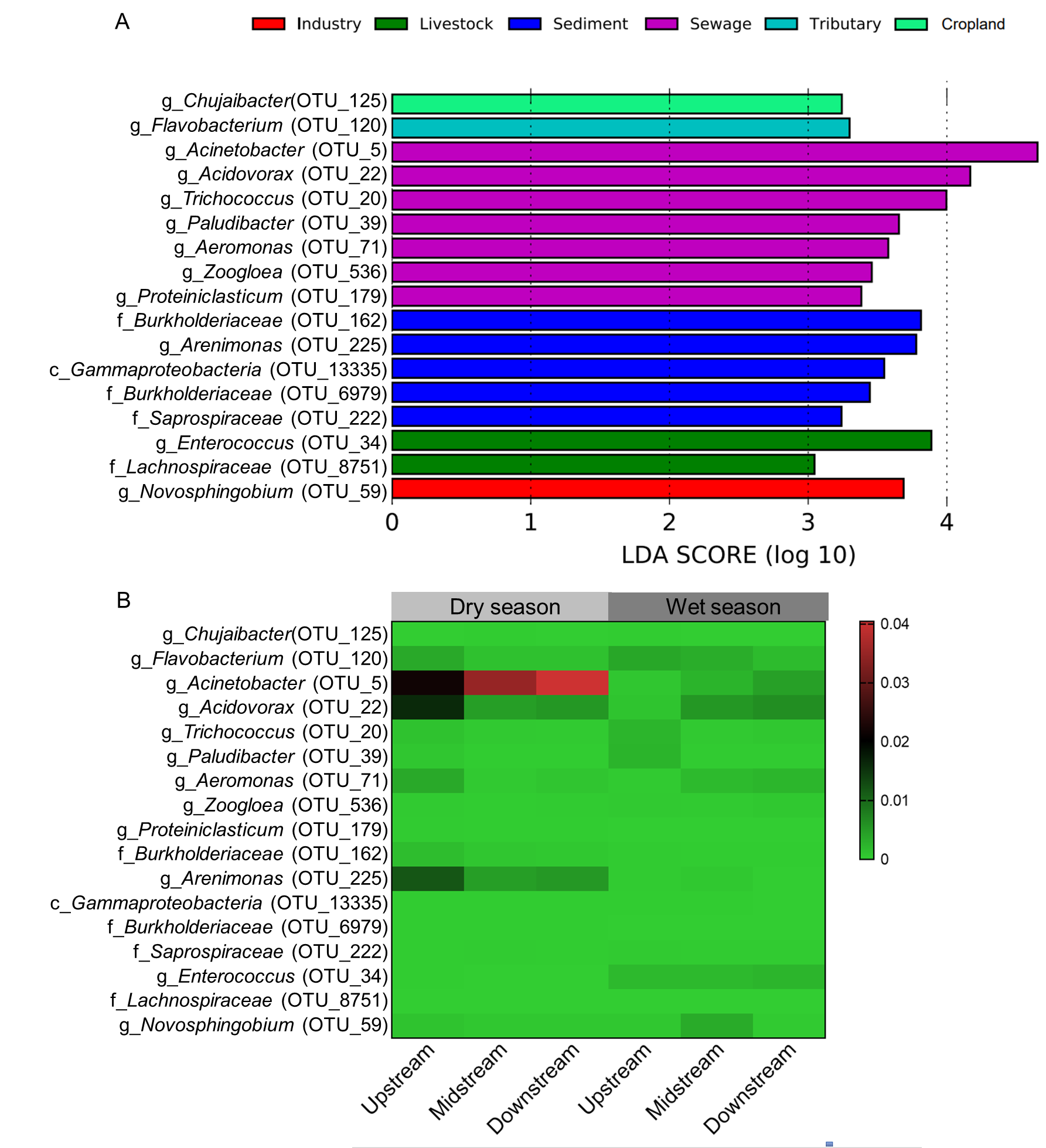


**Supplementary Figure 8** Phylogenetic Mantel correlogram showing significant phylogenetic signal across short phylogenetic distances. Solid and open symbols denote significant and nonsignificant correlations, respectively, relating between-OTU niche differences to between-OTU phylogenetic distances across a given phylogenetic distance. Significantly positive correlations indicate that ecological niche distance between OTUs increases with their phylogenetic distance, but only across the phylogenetic distance class being evaluated (that is, there is phylogenetic signal in OTU environmental niches).


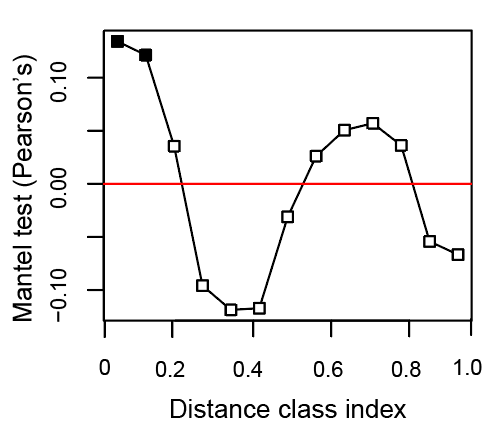


**Supplementary Figure 9** The contribution of ecological processes that governing the assembly of the microbial communities of all the Chishui River samples (A), at the whole Chishui river scale (B), upstream (C), midstream (D) and downstream (E). The percentages (numbers on the individual bars) are given the relative contribution of each known process to the community succession at different river sections.

**
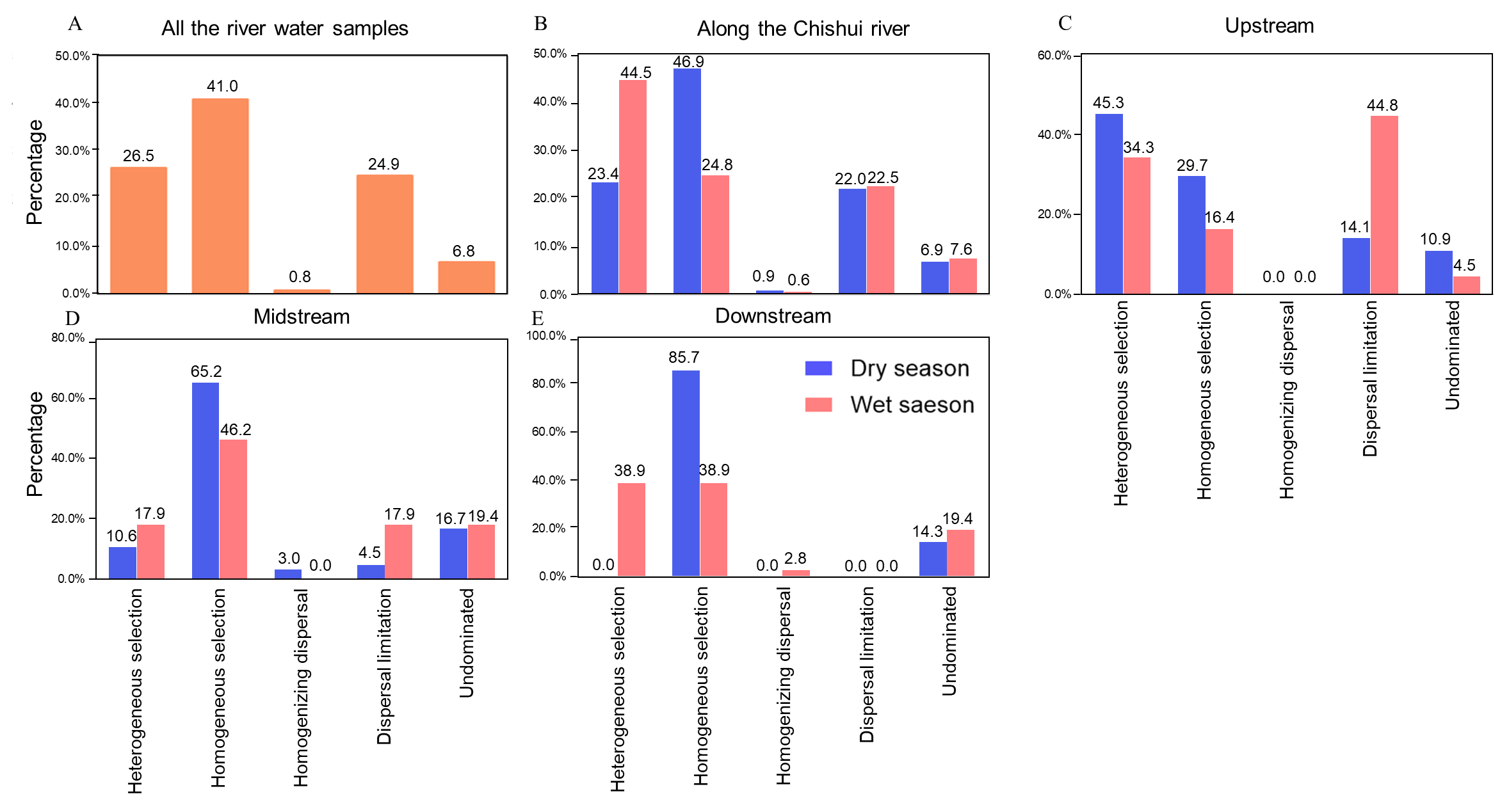
**

**Supplementary table**

**Supplementary Table 1** Permutational multivariate analysis of variance (PERMANOVA) showing the community differences of Chishui River water bacteria.

|  |  | Bray-Curtis | |  | Jaccard | |
| --- | --- | --- | --- | --- | --- | --- |
|  |  | F | p |  | F | p |
| Dry season *vs*  Wet season | Upstream vs Upstream | 12.1596 | 0.001 |  | 5.9849 | 0.001 |
|  | Midstream vs Midstream | 23.0523 | 0.001 |  | 7.4284 | 0.001 |
|  | Downstream vs Downstream | 13.4867 | 0.001 |  | 5.9199 | 0.001 |
| Dry season *vs*  Dry season | Upstream vs Midstream | 11.7357 | 0.001 |  | 2.8823 | 0.001 |
|  | Upstream vs Downstream | 9.8249 | 0.001 |  | 2.3478 | 0.001 |
|  | Midstream vs Downstream | 2.3443 | 0.023 |  | 1.4164 | 0.031 |
| Dry season *vs*  Dry season | Upstream vs Midstream | 3.8315 | 0.001 |  | 1.6295 | 0.013 |
|  | Upstream vs Downstream | 5.6405 | 0.001 |  | 2.444 | 0.001 |
|  | Midstream vs Downstream | 3.1512 | 0.001 |  | 1.7093 | 0.014 |

**Supplementary Table 2** Spearman correlation were used to showed the effects of physiochemical properties on the microbial diversity of the Chishui river water.

|  | Dry season | | | | |  | Wet season | | | | |
| --- | --- | --- | --- | --- | --- | --- | --- | --- | --- | --- | --- |
|  | Shannon | |  | Chao1 | |  | Shannon | |  | Chao1 | |
|  | F | p |  | F | p |  | F | p |  | F | p |
| Turbidity | 0.361 | 0.039 |  | 0.006 | 0.973 |  | 0.421 | 0.018 |  | 0.098 | 0.600 |
| pH | -0.085 | 0.637 |  | 0.119 | 0.510 |  | -0.139 | 0.456 |  | 0.025 | 0.895 |
| Conductivity | -0.618 | 0.001 |  | -0.206 | 0.251 |  | -0.325 | 0.074 |  | -0.030 | 0.873 |
| T | -0.707 | 0.001 |  | -0.509 | 0.002 |  | -0.570 | 0.001 |  | -0.387 | 0.031 |
| DO | 0.411 | 0.018 |  | 0.513 | 0.002 |  | 0.044 | 0.813 |  | 0.201 | 0.277 |
| TN | 0.402 | 0.021 |  | 0.159 | 0.377 |  | 0.247 | 0.180 |  | 0.171 | 0.358 |
| TP | 0.250 | 0.161 |  | 0.076 | 0.675 |  | 0.349 | 0.054 |  | -0.208 | 0.262 |
| COD | -0.081 | 0.656 |  | 0.088 | 0.625 |  | 0.377 | 0.037 |  | -0.151 | 0.419 |
| V | -0.452 | 0.008 |  | -0.477 | 0.051 |  | -0.234 | 0.206 |  | -0.351 | 0.053 |
| Cr | -0.425 | 0.014 |  | -0.344 | 0.051 |  | -0.196 | 0.291 |  | -0.236 | 0.201 |
| Mn | 0.561 | 0.101 |  | 0.515 | 0.002 |  | 0.290 | 0.113 |  | 0.258 | 0.161 |
| Fe | -0.322 | 0.067 |  | -0.162 | 0.367 |  | -0.027 | 0.887 |  | -0.081 | 0.664 |
| Co | -0.410 | 0.018 |  | -0.272 | 0.125 |  | 0.016 | 0.930 |  | -0.073 | 0.696 |
| Ni | 0.107 | 0.554 |  | 0.168 | 0.351 |  | 0.092 | 0.622 |  | 0.023 | 0.904 |
| Cu | -0.250 | 0.160 |  | -0.348 | 0.047 |  | -0.121 | 0.518 |  | -0.204 | 0.271 |
| Zn | 0.167 | 0.353 |  | -0.016 | 0.931 |  | 0.097 | 0.605 |  | 0.051 | 0.788 |
| Cd | 0.104 | 0.565 |  | -0.109 | 0.547 |  | -0.618 | 0.001 |  | -0.455 | 0.010 |
| Sb | -0.269 | 0.129 |  | -0.249 | 0.163 |  | 0.008 | 0.966 |  | -0.030 | 0.873 |
| Ba | -0.580 | 0.001 |  | -0.502 | 0.062 |  | -0.357 | 0.049 |  | -0.355 | 0.051 |
